# The Role of Acceptance in Eating Behaviors—Spanish Validation of Food Craving Acceptance and Action Questionnaire (FAAQ-S)

**DOI:** 10.3389/fpsyg.2021.717886

**Published:** 2021-08-09

**Authors:** Javier Manchón, Maria Quiles, Sofía López-Roig

**Affiliations:** Miguel Hernández University of Elche, Elche, Spain

**Keywords:** eating behaviors, validation, acceptance, food craving, psychological flexibility

## Abstract

**Background:** The Food Craving Acceptance and Action Questionnaire (FAAQ) was developed to measure food craving acceptance, but has not yet been adapted to Spanish. The aim of this study was to validate the FAAQ to the Spanish population and to analyze its psychometric properties.

**Method:** Two studies were conducted. In the first study, the sample consisted of 224 undergraduate students who participated in the comprehension of the Spanish version and the Confirmatory Factor Analysis (CFA). The second sample consisted of 378 participants from a community sample who completed the refined version of the FAAQ and similar and dissimilar measures.

**Results:** Study (1) The CFA was conducted, showing an inadequate fit of the model (CFI = 0.74, RMSEA = 0.18). Therefore, FAAQ was refined and it was administered to the community sample. Study (2) After an exploratory factor analysis, two factors were obtained as in the original FAAQ, Acceptance (30.92% variance explained) and Willingness (36.05%). The internal consistency was adequate for both subscales (ω = 0.88 and ω = 0.87, respectively). Correlation between the factors was *r* = 0.07, which provides evidence that Acceptance and Willingness are different constructs. Correlations of Acceptance with similar variables (*r* between −0.30 and −0.52) were stronger than the dissimilar measures (*r* between −0.26 and 0.24). This did not occur for the Willingness subscale, since correlations were low in all cases (*r* between −0.22 and 0.25).

**Conclusions:** Spanish version of the FAAQ showed evidence of its reliability and validity, and may be a measure to provide a better understanding of how acceptance of thoughts and emotions concerning food and willingness impact eating management behaviors.

## Introduction

Currently, one of the main studied variables to improve the understanding and treatment of eating disorders is food cravings. Food cravings are defined as an intense desire to eat a specific food (Weingarten and Elston, 1991). They are related to binge eating and a wide range of eating disorder pathology (Chao et al., 2016), such as bulimia nervosa (Van den Eynde et al., 2012), and binge eating disorder. Food cravings are also related to obesity and constitute an obstacle to weight loss (Coffino et al., 2018).

Food cravings are often experienced as unwanted. It has been suggested that food cravings may involve intrusive thoughts, and therefore individuals may choose to engage in ingesting food to avoid this unpleasant sensation (Fahrenkamp et al., 2019). In this sense, it seems that the theoretical approach of psychological flexibility from the Acceptance and Commitment Therapy (ACT) could be interesting in the explanation of food cravings and eating behavior (Juarascio et al., 2011). According to this approach, the existence of problematic behavioral patterns could be explained by their rigidity, or lack of psychological flexibility, caused by the attempts to avoid experiencing emotions, sensations, or thoughts perceived as distressing (Hayes et al., 2006). ACT and acceptance-based interventions propose that the acceptance of these internal phenomena and the willingness to experience them while continuing to engage in values-driven behaviors are critical to correctly addressing eating behaviors. Increasing the acceptance and willingness to experience food cravings will allow for the behavioral control necessary to overcome these hedonic drives (Martin et al., 2017) which makes ACT and acceptance-based interventions suitable for addressing eating disorders and weight loss in individuals with obesity.

Although there are general psychological flexibility measures such as the Acceptance and Action Questionnaire II (AAQ-II, Bond et al., 2011), it has been recommended that domain-specific psychological flexibility measures should be used for evaluating particular behaviors (Ong et al., 2019). In order to assess the acceptance and willingness to experience food cravings, the Food Craving Acceptance and Action Questionnaire (FAAQ, Juarascio et al., 2011) was developed. The FAAQ was designed to measure psychological flexibility in a food rich environment, by assessing acceptance of distressing food cravings and willingness to engage in healthy eating even when experiencing food cravings. In the original validation, the FAAQ appeared to be a valid and reliable measure. FAAQ has shown treatment sensitivity and predictive validity detecting pre-post treatment changes and predicting weight loss (Ong et al., 2019). Greater increases in FAAQ appeared to be related to greater weight loss. Interestingly, this correlation was not found with the psychological flexibility as measured by AAQ-II, reflecting the importance of having a domain-specific psychological flexibility instrument (Schumacher et al., 2019).

In the literature, the acceptance of food cravings has been addressed primarily in the understanding of obesity and as a mediator of the acceptance-based behavioral treatment (ABT) in weight loss programs (Forman and Butryn, 2015). In this sense, it has been found that the acceptance of food craving explained food cravings both with direct effect, as well as indirect effect through thought suppression and emotional eating (Coffino et al., 2018). In another study, weight loss maintainers showed greater willingness to ignore food cravings than weight-stable individuals with obesity (Phelan et al., 2020).

The FAAQ has been used to study treatment response in ABT interventions, which contain acceptance strategies as a key element for weight loss. Acceptance-based strategies are able to change eating behavior in the presence of cravings, reducing the consumption of highly palatable foods and decreasing the occurrence of cravings. These strategies appear to be more effective than standard strategies such as cognitive restructuring (Karekla et al., 2020). In weight loss interventions, when compared with standard behavioral treatment (SBT), participants who received ABT lost more weight (Forman et al., 2016) and were about twice as likely to maintain their weight loss after 3 years (Forman et al., 2019). In these studies, food craving acceptance mediated the effect of condition on weight loss and weight regain after treatment. Moreover, it has also been suggested that food craving acceptance may be a moderator of weight loss (Martin et al., 2017). In another ABT intervention for binge eating disorder, it appeared that there were changes in willingness to experiment food cravings related to a reduction in eating pathology (Juarascio et al., 2017).

Although the FAAQ has been used mainly in ACT and ABT interventions, psychological flexibility can be a mechanism of change in numerous types of interventions. An increase in food cravings acceptance was observed whether an ABT or SBT intervention was delivered, although this change occurred to a greater extent in ABT (Schumacher et al., 2019). Ultimately, the FAAQ allows the study of a useful variable for the explanation of eating behavior, providing additional insights that are not present in other variables such as emotional eating or binge eating.

To date, no version of the FAAQ is available in Spanish. The AAQ-II questionnaire is available in Spanish (Ruiz et al., 2013), but there is no validated domain-specific measure such as FAAQ. Moreover, there are instruments that assess state and trait food craving (Spanish versions of the State and Trait Food Cravings Questionnaires, Cepeda-Benito et al., 2000) that could be complemented by the FAAQ. Recent studies showed an increase in the risk of developing eating disorders in the country (e.g., Garrido and Sala, 2015; Parra-Fernández et al., 2018). FAAQ could help in its research by providing a novel variable for a better understanding of this phenomenon. In addition, it could help in the study of the effectiveness of ACT interventions that are already being implemented (Marco et al., 2018). Therefore, the aim of this study is to adapt the Spanish version of Food Craving Acceptance and Action Questionnaire and to analyze its psychometric properties. It is expected to reproduce the two-factor structure (acceptance and willingness), and that the FAAQ in its Spanish version will be a reliable and valid measure.

## Method

### Participants

In this work, two studies were conducted with their respective samples. The characteristics of both samples are included in Table 1. Sample 1 consisted of 232 undergraduate students and Sample 2 consisted of 378 participants from a community sample.

**Table 1:** Study samples characteristics.

	**Sample 1**	**Sample 2**
Mean age in years (*SD*)	22.31 (3.85)	31.77 (14.46)
Age range	18–45	18–73
Gender	75.9% women	70.9% women
Mean BMI in kg/m^2^ (*SD*)	22.41 (3.43)	23.25 (4.26)
BMI range	16.4–35.2	16.1–49.6
**BMI classification**		
% Underweight	8.6%	9.6%
% Normal weight	72.0%	61.9%
% Overweight	15.5%	22.4%
% Obese	3.9%	6.1%

Regarding the calculation of the sample size, for Sample 1 it was taken into account that a Confirmatory Factor Analysis (CFA) would be performed. In accordance with a 20:1 *N:q* ratio, and considering that the data were ordinal, the minimum sample size required was 200 cases (Kyriazos, 2018).

In Sample 2, it was taken into account that an Exploratory Factor Analysis (EFA) would be performed. We expected to find communalities greater than 0.40 with a two-factor structure, having a minimum of 4 items per factor, so the minimum sample size required was 200 cases (Lloret-Segura et al., 2014).

### Instruments

The Food Craving Acceptance and Action Questionnaire (FAAQ; Juarascio et al., 2011). It was constructed to measure the acceptance of food cravings. The original version was based on the Chronic Pain Acceptance Questionnaire (CPAQ; McCracken et al., 2004). The final version consisted of 10 items that were scored on a Likert-type scale with a range from 1 (Almost never true) to 6 (Always true). The final score is calculated by summing the 10 items after reversing the scores of negative items (3, 4, 6, 7, and 9). Higher scores suggest greater acceptance of food cravings. The internal consistency of the questionnaire was measured on several occasions in the original study, obtaining, as a result, Cronbach's α = 0.66, α = 0.68, and α = 0.93. Regarding its factorial structure, two factors emerged explaining 62.48% of the variance. The first factor, Willingness, was comprised by items 1, 2, 3, 5, 8 and 10, with a range of factor saturations between 0.59 and 0.82, and an internal consistency of Cronbach's α = 0.82. Willingness reports the extent to which a person may engage in behaviors consistent with one's own values for weight management and healthy eating despite the fact that it may cause unpleasant thoughts and feelings such as cravings. The second factor, Acceptance, was comprised by items 4, 6, 7, and 9 with a range of factor saturations between 0.74 and 0.87, and an internal consistency of Cronbach's α = 0.60. Acceptance reflects the degree to which a person is open to experiencing cravings, emotions, and physiological experiences associated with food without attempts to control, alter, suppress, or avoid these experiences.

The Acceptance and Action Questionnaire—II (AAQ-II; Bond et al., 2011; Spanish version by Ruiz et al., 2013). It is a general measure of psychological flexibility. In this study, the internal consistency was Cronbach's α = 0.93.

The Three-Factor Eating Questionnaire R-18 (TFEQ-R18; Stunkard and Messick, 1985; Spanish version by Jáuregui-Lobera et al., 2014). This scale measures three factors of eating behaviors, these being cognitive restraint, uncontrolled eating and emotional eating. In this study, the internal consistency was Cronbach's α = 0.89.

The Hospital Anxiety and Depression Scale (HADS; Zigmond and Snaith, 1983; Spanish version by Terol-Cantero et al., 2007). It consists of 14 items in two subscales measuring anxiety and depression, and a total score that evaluates global psychological distress. In this study, the internal consistencies were Cronbach's α = 0.72 and α = 0.84 for the anxiety and depression subscales.

The Rosenberg Self-Esteem Scale (RSE; Rosenberg, 1979; Spanish version by Atienza et al., 2000). This instrument assesses self-esteem, understood as feelings of personal worth and self-respect. In this study, the internal consistency was Cronbach's α = 0.91.

#### Body Mass Index

This index (kg/m^2^) was obtained from self-reported weight (kg) and height (m).

### Procedure

First, the Research Ethics Committee of the university approved the project and the data collection.

The translation and cultural adaptation procedure was based on López-Roig and Pastor (2016). Two of the authors translated the FAAQ items independently. The two resulting versions were compared for consensus. Later, the back-translation was carried out by two bilingual speakers (Spanish and English). These translations were compared with the original version to resolve possible errors in the adaptation. Once this procedure was completed, 30 undergraduate students completed the questionnaire in order to guarantee the comprehension of the items. After analyzing the students' opinions, minor wording changes were made to three of the items, while item 5 was rephrased because it showed comprehension difficulties.

For the first study, the questionnaires were administered in the university classrooms during academic hours, with prior agreement with the lecturers. Students were asked to complete the questionnaire after reading the participant information sheet. If they agreed to participate, they signed the informed consent and completed the self-administered questionnaire. No financial or academic compensation of any kind was offered. Missing values were imputed by calculating the median of the item in the sample, in those cases in which missing values did not exceed the 20% of the items in the instrument. Otherwise, all the questionnaire scores were removed.

For the second study, the questionnaires were administered online due to COVID-19 restrictions. The paper-and-pencil or online administration of the questionnaires does not seem to imply differences in scores, as shown in previous studies (e.g., Van de Looij-Jansen and De Wilde, 2008; Ward et al., 2014). Sampling was carried out by sharing a link on social networks (WhatsApp, Instagram, and email) to general population. The participant information sheet and the consent form were available prior to the study. If they agreed to participate, they signed the informed consent and completed the questionnaires. No economic compensation of any kind was offered. The response to the items was mandatory to submit the questionnaires, so there were no missing values.

### Data Analysis

The statistical computing R environment 4.0.1 was used for the data analyses. First, a CFA was conducted in order to replicate the factorial structure found by Juarascio et al. (2011) with the data from the Sample 1. For this purpose, the lavaan package (Rosseel, 2012) was used. The method of parameter estimation was DWLS (diagonally weighted least squares) since data were ordinal and non-normally distributed (Li, 2015). The indices used for testing the model fit and the expected values were the chi-square test, the comparative fit index (CFI > 0.95), the Tucker-Lewis index (TLI > 0.95), the root mean square error of approximation (RMSEA < 0.06), and the standardized root mean-square residual (SRMR < 0.08). These criteria (Hu and Bentler, 1999), however, should be taken with caution since the DWLS estimation tends to overestimate the model fit and yield lower RMSEA values (Xia and Yang, 2019). Particular attention was paid to the SRMR since it is a robust indicator regardless of the method of estimation (Shi and Maydeu-Olivares, 2020). R's psych package (Revelle, 2020) was used to perform the descriptive analyses, internal consistency values (Cronbach's α and McDonald's ω coefficients), Pearson correlations, Fisher's *Z* transformation to compare correlations and EFA in Sample 2. For the EFA, the KMO index was calculated, which must exceed 0.80 to show an adequate fit (Lloret-Segura et al., 2014).

## Results

### Study 1

First, a CFA was performed following the original validation. Therefore, the items were divided into the factors of Acceptance and Willingness. The model fit was not adequate, presenting unacceptable estimates of error (χ^2^_[34]_ = 384.99, *p* < 0.001; CFI = 0.74, TLI = 0.66, RMSEA = 0.18 [0.16 ~0.20 CI 90%], SRMR = 0.17). According to the analysis, the Acceptance factor presented an adequate structure with parameter estimates between 0.58 and 0.75. This was not the case for the Willingness factor, that ranged between 0.01 (item 8) and 0.78 (item 3). The parameter estimates can be found in Table 2.

**Table 2:** Descriptive statistics of the items, item-factor correlations, CFA parameter estimates, and internal consistency.

	***M* (*SD*)**	**Item-factor *r***	**CFA parameter estimates**	**α**	**ω**
*Willingness*				0.53	0.63
FAAQ1	3.75 (1.36)	0.45	0.10		
FAAQ2	4.06 (1.34)	0.30	−0.42		
FAAQ3	3.97 (1.56)	−0.02	−0.78		
FAAQ5	3.70 (1.49)	0.01	0.41		
FAAQ8	3.92 (1.38)	0.57	0.01		
FAAQ10	4.21 (1.37)	0.54	−0.04		
*Acceptance*				0.79	0.80
FAAQ4	4.46 (1.45)	0.59	0.75		
FAAQ6	3.29 (1.61)	0.51	0.58		
FAAQ7	4.12 (1.66)	0.67	0.72		
FAAQ9	3.92 (1.66)	0.66	0.74		

In order to detect possible problems in the items, internal consistency and item-factor correlations were obtained (Table 2). The internal consistency of the scale was α = 0.69 (ω = 0.73), and it increased to α = 0.76 (ω = 0.77) when item 5 was removed. Regarding the internal consistency of the factors, they were α = 0.79 (ω = 0.80) for Acceptance and α = 0.53 (ω = 0.63) for Willingness. In order to obtain an acceptable internal consistency in Willingness (α = 0.80, ω = 0.81), it was necessary to remove items 3, 5, and 2. At this point, the CFA was repeated without items 2, 3, and 5, yielding unacceptable results in RMSEA and SRMR (χ^2^_[13]_ = 48.42, *p* < 0.001; CFI = 0.95, TLI = 0.92; RMSEA = 0.10 [0.07 ~0.12 CI 90%], SRMR = 0.08).

In view of the results of the CFA and the internal consistency in the Willingness subscale, the questionnaire was modified. For this purpose, it was decided to adapt several items from the CPAQ (McCracken et al., 2004).

### Study 2

First, it was decided to drop item 5 when refining the questionnaire. This item presented comprehension problems and had a negative item-total correlation that affected the internal consistency of the scale. In second place, based on the CPAQ (McCracken et al., 2004), and following (Juarascio et al., 2011) item construction process, four items of the CPAQ were adapted by changing the reference to chronic pain to healthy eating or dieting. These were the items 5 (“I am committing to healthy eating habits no matter how unpleasant or demanding I may find it”), 11 (“It's okay to experience unpleasant feelings while dieting”), 12 (“I can maintain my commitment to healthy eating even when I am involved with other responsibilities”), and 13 (“When I start to feel like giving up my healthy eating habits, I find a way to continue to do them”). Prior to administering the questionnaire, the process of translation, back-translation and comprehension of these four items was repeated.

An EFA was performed reproducing the conditions of the original validation by Juarascio et al. (2011). Therefore, the EFA was performed with generalized least squares and oblique rotation. In a first phase of analysis, a three-factor solution was obtained (KMO = 0.84; 65.32% variance explained). However, the factor loadings of items 2, 5, and 11 were very similar in all three factors. They were close to 0 in items 2 and 11, and between 0.43 and 0.52 in the case of the item 5. The EFA was repeated removing these three items. This second analysis showed a two-factor solution, explaining 66.97% of the variance (KMO = 0.82). It was therefore decided to exclude these three items. The Acceptance factor explained 30.92% of the variance, while Willingness explained the 36.05%. The factor loadings of the items, as well as their mean scores and standard deviations of the final solution are shown in Table 3. It should be noted that the item 3, originally allocated in the Willingness factor, appeared in the Acceptance factor. Therefore, Acceptance contained items 3, 4, 6, 7, and 9. The remaining original items of the Willingness factor (1, 8, and 10), as well as two of the new items (12 and 13) loaded together. After analyzing item 3, it theoretically reflected contents congruent with Acceptance.

**Table 3:** Exploratory factor analysis loadings and descriptive statistics of the refined version of the FAAQ.

**Items**	**Factor 1** ** Willingness**	**Factor 2** ** Acceptance**	***M* (*SD*)**
1. Sigo una alimentación saludable aun cuando tengo el deseo de comer en exceso o de escoger alimentos no sanos.*I continue to eat a healthy diet, even when I have the desire to overeat or make poor eating choices*.	**0.75**	−0.05	3.73 (1.44)
3. Es necesario que controle mis impulsos por comer para cuidar mi alimentación.*It's necessary for me to control my food urges in order to control my eating*.	−0.02	**0.78**	3.78 (1.65)
4. Necesito concentrarme en eliminar mi impulso por comer de manera no saludable.*I need to concentrate on getting rid of my urges to eat unhealthily*.	0.24	**0.80**	4.24 (1.68)
6. Controlar mis impulsos por comer poco saludable es tan importante como controlar mi alimentación.*Controlling my urges to eat unhealthily is just as important as controlling my eating*.	−0.12	**0.68**	3.38 (1.66)
7. Mis pensamientos y sentimientos con respecto a la comida deben cambiar antes de poder hacer cambios en mi alimentación o dieta.*My thoughts and feelings about food must change before I can make changes in my eating*.	0.16	**0.77**	3.99 (1.69)
8. A pesar de tener antojos por alimentos poco saludables, sigo comiendo sano.*Despite my cravings for unhealthy foods, I continue to eat healthily*.	**0.82**	−0.02	3.89 (1.44)
9. Antes de poder hacer un cambio alimentario importante, tengo que tener cierto control sobre mis impulsos alimentarios.*Before I can make any important dietary changes, I have to get some control over my food urges*.	0.02	**0.77**	3.65 (1.67)
10. Aunque tenga el deseo de comer algo poco saludable, soy capaz de comer sano.*Even if I have the desire to eat something unhealthy, I can still eat healthily*.	**0.75**	0.09	4.21 (1.43)
12. Puedo mantener mi compromiso de comer sano incluso cuando estoy ocupado/a con otras responsabilidades.“*I can maintain my commitment to healthy eating even when I am involved with other responsibilities.”*	**0.78**	0.12	3.99 (1.46)
13. Cuando empiezo a sentir deseos por abandonar mis hábitos alimentarios saludables, encuentro una manera de continuar realizándolos.“*When I start to feel like giving up my healthy eating habits, I find a way to continue to do them.”*	**0.79**	0.10	3.78 (1.42)

*Note: The English items in italics are the original items. The English items in quotation marks are the translation of the new items generated in Spanish. The numbers in bold indicate the inclusion in that factor*.

Once the final factorial solution was obtained, internal consistency and item-factor correlations were calculated. For the Willingness factor, the internal consistency was α = 0.88 (ω = 0.88) and its item-factor correlations were between 0.68 and 0.75. In the case of Acceptance, the internal consistency was α = 0.86 (ω = 0.87) and its item-factor correlations were between 0.62 and 0.71. The internal consistency for the total score was α = 0.80 (ω = 0.80), and the item-scale correlations were between 0.33 and 0.57. The correlation between Acceptance and Willingness was nearly zero (*r* = 0.07). As for the correlations with the total score, Acceptance showed a correlation of *r* = 0.77, and *r* = 0.69 in the case of Willingness.

Next, a correlation analysis (Table 4) was performed to examine convergent and discriminant validity, following the considerations employed by Juarascio et al. (2011). The authors made a comparison between the sums of similar constructs vs. different variables, so the hypothesized result was that the sum of correlations of similar constructs would be greater than that of different variables. The difference in the sum of correlations was observed in the Total score (Σ*r* = |0.36| in similar variables vs. Σ*r* = |0.25| in discriminant constructs, *Z* = 1.66, *p* < 0.05) and in Acceptance (Σ*r* = |0.44| vs. Σ*r* = |0.23|, *Z* = 3.25, *p* < 0.01). No such differences were found in the Willingness factor (Σ*r* = |0.17| vs. Σ*r* = |0.15|, *Z* = 0.28, *p* = 0.39).

**Table 4:** Correlations between the FAAQ and other variables.

	**FAAQ Total**	**FAAQ Acceptance**	**FAAQ Willingness**
*Similar*			
AAQ-II	−0.30^**^	−0.30^**^	−0.14^**^
TFEQ Desinhibition	−0.47^**^	−0.51^**^	−0.17^**^
TFEQ Emotional eating	−0.52^**^	−0.52^**^	−0.21^**^
TFEQ Restraint	−0.18^**^	−0.47^**^	0.25^**^
BMI	−0.27^**^	−0.34^**^	−0.05
*Dissimilar*			
HADS Total	−0.28^**^	−0.25^**^	−0.16^**^
HADS Anxiety	−0.14^**^	−0.16^**^	−0.05
HADS Depression	−0.32^**^	−0.26^**^	−0.22^**^
RSE	0.28^**^	0.24^**^	0.16^**^

*AAQ-II, Acceptance and Action Questionnaire—II; TFEQ, Three Factor Eating Questionnaire; BMI, Body Mass Index; HADS, Hospital Anxiety and Depression Scale; RSE, Rosenberg Self-Esteem Scale;*

** *statistically significant at p < 0.01*.

## Discussion

The purpose of this work was the adaptation of the Food Craving Acceptance and Action Questionnaire to the Spanish population. After the two studies presented, it can be stated that the FAAQ appeared to be a reliable and valid instrument. With this work, we intended to provide an instrument for the evaluation of the acceptance of food cravings for its use in Spain.

First, the CFA was performed, which showed an inadequate factor solution. Moreover, internal consistency problems were detected in half of the items of Willingness (2, 3, and 5), but their exclusion from the model failed to provide a satisfactory factorial structure. Overall, the CFA and the reliability results allowed to identify weaknesses in the Willingness factor. Interestingly, Willingness showed better results in the original work by Juarascio et al. (2011) in terms of internal consistency, while in this case Acceptance showed adequate psychometric properties in both the CFA and reliability.

As commented above, it was decided to drop item 5. This item presented comprehension problems and had a negative item-total correlation that affected the internal consistency of the scale. A possible explanation for these results can be found in the translation and subsequent difficulties in the comprehension of the item.

After the new items based on the CPAQ were included, an EFA was carried out in which five items were retained in both factors. Item 2, which showed psychometric deficiencies in both studies, was definitely discarded. In the case of item 3, it appeared as part of the Acceptance factor. As mentioned above, the content of this item refers to the control of food urges. This same content is reflected in other items of Acceptance, and therefore item 3 had theoretical consistency with the rest of the items. Considering the addition of new items, the EFA yielded two factors that showed adequate internal consistency and factor structure. This addition solved the performance of Willingness, while Acceptance continued to show adequate psychometric properties. The new items could also explain that Acceptance and Willingness appear to be orthogonal factors since the correlation between them was near to zero. This result differs from the original questionnaire (Juarascio et al., 2011).

Given this factorial solution, the Acceptance items content referred to the elimination, control, or modification of thoughts or urges related to food craving. This content would correspond with the lack of acceptance as explained in the literature (Hayes et al., 2006), emphasizing that these experiences are perceived as unwanted (Fahrenkamp et al., 2019).

Also, Willingness items would be closer to ACT's values variable, which involves giving purpose to behaviors. Through the work on values, individuals focus on increasing the ability to live a meaningful life together with the discomfort of challenges (Dahl, 2015). The Willingness factor would indicate a disposition or commitment to maintain healthy eating in spite of the appearance of urges to eat (Martin et al., 2017). It might be interesting to study the correlation between FAAQ and other instruments such as the Valuing Questionnaire (Smout et al., 2014) since higher correlations than the aforementioned in this paper might appear.

Regarding convergent and divergent validity, the results pointed to Acceptance having a statistically greater relationship with similar variables than with theoretically dissimilar variables. This did not occur with the Willingness factor. In this case, a contrast was found with the results of Juarascio et al. (2011). In their results, it was the opposite for the two factors. In our view, the fact that Willingness did not present the expected correlations does not undermine the validity of the FAAQ. Rather, it would suggest that it is a different construct, and this can be supported by the null correlation between factors.

Correlations with BMI were significant with FAAQ total score and Acceptance. This relation is meaningful since in an ABT weight loss intervention it was found that the change through the intervention in FAAQ scores mediated the intervention effect at 24 months in weight loss (Forman et al., 2019). This implies that the FAAQ might be of high interest for future research in weight management.

With respect to the correlation with the AAQ-II, it was of moderate intensity, which confirms the relevance of having a domain-specific measure of psychological flexibility as mentioned above (Ong et al., 2019).

Regarding its limitations, first, the CFA sample consisted only of undergraduate students. Since it was a homogeneous sample, there may have been bias in the results. Although the sample size was adequate in both studies according to the statistical requirements, it would be advisable to include larger samples in future studies. The FAAQ should also be tested in a clinical sample. As reported by Juarascio et al. (2011) and Ong et al. (2019), FAAQ was able to detect clinical changes in weight loss, so it should be proven to be useful for that purpose. In addition, the samples were mostly composed of women, so more men should be included in future studies. In relation to other psychometric analyses, it would be convenient to test temporal stability and measurement invariance (e.g., gender).

The FAAQ has shown adequate psychometric properties in its Spanish version. The use of third-wave psychological therapies such as ACT for eating disorders in Spain (Marco et al., 2018) make it necessary to have valid and reliable measures of their related variables. This measure may help to better understand the relationship between acceptance and problematic eating behaviors such as binge eating or emotional eating, and to better understand the mechanisms of action of the efficacy of weight management interventions in the Spanish research context.

## Data Availability Statement

The datasets presented in this article are not readily available because participants of this study did not agree for their data to be shared publicly, so supporting data is not available. Requests to access the datasets should be directed to Maria Quiles, mj.quiles@umh.es.

## Ethics Statement

The studies involving human participants were reviewed and approved by Oficina de Investigación Responsable from Miguel Hernández University of Elche. The patients/participants provided their written informed consent to participate in this study. The patients/participants provided their written informed consent to participate in this study.

## Author Contributions

JM, MQ, and SL-R designed the study and carried out the process of adaptation into Spanish. JM and MQ collected the data and wrote the manuscript. JM analyzed the data. SL-R gave feedback on the manuscript. All authors approved the final version.

## Conflict of Interest

The authors declare that the research was conducted in the absence of any commercial or financial relationships that could be construed as a potential conflict of interest.

## Publisher's Note

All claims expressed in this article are solely those of the authors and do not necessarily represent those of their affiliated organizations, or those of the publisher, the editors and the reviewers. Any product that may be evaluated in this article, or claim that may be made by its manufacturer, is not guaranteed or endorsed by the publisher.
